# Diagnostic use of lung ultrasound compared to chest radiograph for suspected pneumonia in a resource-limited setting

**DOI:** 10.1186/s12245-018-0170-2

**Published:** 2018-03-12

**Authors:** Yogendra Amatya, Jordan Rupp, Frances M. Russell, Jason Saunders, Brian Bales, Darlene R. House

**Affiliations:** 1 Department of General Practice and Emergency Medicine, Patan Academy of Health Sciences, PO Box 26500, Kathmandu, Nepal; 2 Department of Emergency Medicine, Vanderbilt University Medical Center, Nashville, TN USA; 3 Department of Emergency Medicine, Indiana University School of Medicine, Indianapolis, IN USA

**Keywords:** Lung ultrasound, Pneumonia, Nepal, Diagnosis, Developing countries

## Abstract

**Background:**

Lung ultrasound is an effective tool for diagnosing pneumonia in developed countries. Diagnostic accuracy in resource-limited countries where pneumonia is the leading cause of death is unknown. The objective of this study was to evaluate the sensitivity of bedside lung ultrasound compared to chest X-ray for pneumonia in adults presenting for emergency care in a low-income country.

**Methods:**

Patients presenting to the emergency department with suspected pneumonia were evaluated with bedside lung ultrasound, single posterioranterior chest radiograph, and computed tomography (CT). Using CT as the gold standard, the sensitivity of lung ultrasound was compared to chest X-ray for the diagnosis of pneumonia using McNemar’s test for paired samples. Diagnostic characteristics for each test were calculated.

**Results:**

Of 62 patients included in the study, 44 (71%) were diagnosed with pneumonia by CT. Lung ultrasound demonstrated a sensitivity of 91% compared to chest X-ray which had a sensitivity of 73% (*p* = 0.01). Specificity of lung ultrasound and chest X-ray were 61 and 50% respectively.

**Conclusions:**

Bedside lung ultrasound demonstrated better sensitivity than chest X-ray for the diagnosis of pneumonia in Nepal.

**Trial registration:**

ClinicalTrials.gov, registration number NCT02949141. Registered 31 October 2016.

## Background

Pneumonia is one of the leading causes of death worldwide, and in low-income countries, it is the leading cause of death [1]. These deaths may be prevented by early detection and targeted antibiotic therapy [2]. However, the diagnosis of pneumonia is not always clear on presentation to health care facilities. Imaging usually includes a chest X-ray or, in some cases, a thoracic computed tomography (CT) scan. While the latter has the highest sensitivity, it is associated with high costs and higher doses of radiation [3, 4]. Because of these limitations, chest X-ray continues to be the main diagnostic modality for pneumonia despite its low sensitivity (43–78%) [4–8].

Several studies in high-income countries and one study by Liu et al. in China, a middle-income country, have shown ultrasound to be a reliable tool to diagnose pneumonia with a higher sensitivity and specificity than chest X-ray [8–13]. Ultrasound is a safe, portable, and inexpensive diagnostic modality that is widely used in resource-limited countries [14]. In remote areas with limited access to X-rays, clinicians are reliant on their physical examination, which also has limited sensitivity (47–69%), and therefore, ultrasound for diagnosing pneumonia has the potential to be very useful in this setting [15]. Even in urban settings, use of bedside ultrasound can alleviate diagnostic dilemmas with critically ill patients, especially where facilities lack portable X-rays. However, the diagnostic accuracy of ultrasound to diagnose pneumonia in low-income countries like Nepal has not yet been studied. Results from studies in high-income countries are difficult to extrapolate to settings like Nepal where there are not only higher rates of pneumonia, but also high rates of tuberculosis (245 per 100,000) and chronic obstructive pulmonary disease (estimated 23–43% prevalence), which may make clinical and radiographic diagnosis of pneumonia more difficult [16–20].

Therefore, the objective of this study was to evaluate the sensitivity of bedside lung ultrasound compared to chest X-ray for the diagnosis of pneumonia in patients presenting to the emergency department in Nepal.

## Methods

### Study design

A prospective, convenience sample of patients presenting to the emergency department with suspected pneumonia were enrolled when a trained sonographer was present. Patients were enrolled from November 2016 to March 2017. This study was approved by both Vanderbilt University Institutional Review Board and Nepal Health Research Council Ethical Review Board. The study was registered with ClinicalTrials.gov, registration number NCT02949141. Written informed consent in Nepali was obtained from patients prior to enrollment. Lung ultrasounds and chest CT scans were provided free of charge to the patient.

### Study setting and population

Located in the Kathmandu Valley, Patan Hospital is a large urban hospital with a 35-bed emergency department. The emergency department has an annual volume of approximately 36,000 patients with a 20% admission rate.

We included patients age 18 or older with suspected pneumonia. Patients had at least three of the following signs or symptoms: temperature greater than 38 °C or history of fever, cough, dyspnea, tachypnea (respiratory rate greater than 20), or oxygen saturation lower than 92%.

### Study protocol

Four emergency department faculty and resident physicians were trained on the use of lung ultrasound for diagnosing pneumonia. This training included a 1-h lecture on lung ultrasound followed by a week of performing lung ultrasounds in the department. Prior to enrollment, clinicians each performed five lung ultrasounds with interpretations. These pre-enrollment scans were reviewed by an expert sonographer with registered diagnostic medical sonographer certification to ensure adequate skill in acquiring and interpreting lung ultrasounds. Prior to enrollment, a kappa analysis revealed good inter-rater reliability between the trainees and the expert sonographer (*k* = 0.67).

Once consent was obtained, the clinician recorded patient demographics, symptoms, and physical examination findings on a standardized data collection sheet. After clinical evaluation, a bedside lung ultrasound was performed. Sonographers were not blinded to clinical information. A Sonosite M Turbo (Fujifilm Sonosite, Inc.) ultrasound machine with a curvilinear probe was used. In accordance with previous literature, the ultrasound examination included ten views: two anterior views and two lateral views (one including the costophrenic angle), and one posterior view on each hemithorax [10, 21]. The physician recorded ultrasound findings and interpretation. An ultrasound diagnosis of pneumonia was defined as the presence of unilateral B lines or subpleural lung consolidation.

All patients had a single posterioranterior (PA) chest X-ray as a part of the standard evaluation. Two-view PA and lateral chest X-rays are not routinely ordered in this setting as it requires patients to pay for a second X-ray. For this reason, ultrasound was compared to the single PA view only. Clinicians performing the lung ultrasound were blinded to the results of the chest X-ray. The chest X-ray was read by a board-certified radiologist, who was blinded to the clinical presentation and the results of any other imaging. Patients then underwent chest CT, as the diagnostic standard to evaluate for pneumonia. The CT was also read by a radiologist who was blinded to the clinical presentation and results of the previous studies. If the radiology read was indeterminate, the radiologist was asked specifically regarding the presence of pneumonia and the response was recorded. Chest X-ray and CT interpretations were collected for statistical analysis.

### Data analysis

Sensitivity, specificity, positive and negative predictive values, and likelihood ratios for both lung ultrasound and chest X-ray were calculated. According to McNemar’s test comparing proportions of paired samples, a sample size of 62 patients was needed to detect a 20% difference between chest X-ray and lung ultrasound based on a sensitivity of chest X-ray of 70%. Using R statistical program (version 3.3.2), McNemar’s test was used to evaluate any statistical difference in sensitivity between chest X-ray and lung ultrasound. A kappa analysis was done to determine inter-rater reliability for ultrasound interpretations between the clinician sonographer and an expert sonographer with registered diagnostic medical sonographer certification. The expert sonographer was blinded to the clinician’s interpretation and results of other imaging.

## Results

Sixty-two patients were enrolled in the study. Demographic information is presented in Table 1.

**Table 1:** Patient demographics

	Pneumonia (*n* = 44)	No pneumonia (*n* = 18)
Age range, year		
30–39	3 (7)	2 (11)
40–49	9 (20)	3 (17)
50–59	10 (23)	3 (17)
60–69	13 (30)	4 (22)
70–79	6 (14)	2 (11)
≥ 80	3 (7)	4 (22)
Mean Age (SD)	58.5 (13.8)	61.2 (16.3)
Women	26 (59)	7 (39)
History of chronic obstructive pulmonary disease	18 (41)	11 (61)
History of tuberculosis	4 (9)	1 (6)

Values are presented as number (%)

Forty-four (71%) patients had pneumonia based on CT. Ultrasound was positive in 40 of the 44 patients with pneumonia, demonstrating a sensitivity of 91%. Chest X-ray was positive in 32 of the 44 patients with pneumonia, yielding a sensitivity of 73% (Table 2). The sensitivity of ultrasound was significantly better than chest X-ray (*p* = 0.01). Specificity of ultrasound and chest X-ray were similar at 61 and 50% respectively (*p* = 0.62). The positive predictive value of lung ultrasound was 85% and chest X-ray was 78%. The negative predictive value of ultrasound was 73% while chest X-ray was 43%. The positive likelihood ratio for diagnosing pneumonia with lung ultrasound was 2.34, while the negative likelihood ratio was 0.15. Chest X-ray had a positive and negative likelihood ratio of 1.45 and 0.55 respectively.

**Table 2:** Results of chest X-ray and lung ultrasound compared with CT

	CT positive (*n* = 44)	CT negative (*n* = 18)
Chest X-ray		
Positive	32	9
Negative	12	9
Ultrasound		
Positive	40	7
Negative	4	11

In patients with a false-positive ultrasound, CT diagnoses were the following: bronchiectasis with fibrosis (*n* = 3), interstitial lung disease (*n* = 2), tuberculosis (*n* = 1), and normal (*n* = 1). In the one patient with a normal CT scan, lung ultrasound was positive based on B lines in the posterior zone only.

Inter-rater reliability between ultrasound interpretations by the sonographer and expert reviewer was 0.79, demonstrating very good agreement.

Clinicians performed lung ultrasound in an average of 7 min 9 s (SD 1 min 57 s). The time it took for patients to get a chest X-ray was 117 min (SD 56 min). This time did not include the time required for film printing and pickup for clinician review.

## Discussion

To our knowledge, this is the first study to provide evidence that the sensitivity of lung ultrasound is superior to chest X-ray for diagnosing pneumonia in a low-income country.

Lung ultrasound demonstrated a higher sensitivity for the diagnosis of pneumonia compared to chest X-ray. Previous studies have found similar results regarding the sensitivity of ultrasound for pneumonia. In recent meta-analyses, Long et al. found a pooled sensitivity of 88% for lung ultrasound and Ye et al. found a pooled sensitivity of 95% [7, 22]. However, in these meta-analyses, the majority of studies used biased reference standards such as chest X-ray and hospital discharge diagnoses, which may confound their results. Using similar methods to our study with CT as the reference standard, Liu et al.’s study in China found similar results with the sensitivity of lung ultrasound significantly outperforming chest X-ray (94.6 versus 77.7%, *p* < 0.001) [8].

Ultrasound missed pneumonia in four patients. These pneumonias were also missed by chest X-ray. In all four patients, the pneumonia was located in the middle of the lung parenchyma and did not extend to the pleura. This is similar to prior studies finding that lesions not extending to the pleura are missed by ultrasound [11]. Therefore, clinicians should continue to consider close follow-up for repeat evaluation in patients with continued high suspicion for pneumonia and negative lung ultrasound.

Specificities of lung ultrasound and chest X-ray for pneumonia were similar. Lung ultrasound specificity was significantly lower than previous studies, including the two meta-analyses previously mentioned. Long et al. found a pooled specificity of 86% while Ye et. Al found a pooled specificity of 91% [7, 22]. Using CT as the gold standard, Liu et al. found a specificity of lung ultrasound to be significantly better than chest X-ray (99 versus 61.1%, *p* < 0.001) for the diagnosis of pneumonia [8]. While this study was done in China, where there are also high rates of tuberculosis and chronic obstructive pulmonary disease, there were no patients with tuberculosis in the study and only 10% of patients had a diagnosis of chronic obstructive pulmonary disease, making it difficult to extrapolate these results to other areas of China and other middle- and low-income countries. The low specificity for ultrasound in our study is due to a higher prevalence of co-morbidities, including previous pulmonary tuberculosis, bronchiectasis, and chronic obstructive lung disease. Patients with history of tuberculosis often develop significant parenchymal scarring and fibrosis with bronchiectasis [23]. The CT findings for the majority of the false positives demonstrated significant chronic lung disease with fibrosis that made ultrasound findings consistent with consolidation [24]. Clinically, these false positives may not impact clinical care as patients with chronic lung disease may also benefit from antibiotic use in acute exacerbations [25]. However, similar to chest X-ray, low specificity may lead to over-diagnosis of pneumonia and inappropriate use of antibiotics.

One false-positive ultrasound was in a patient with active pulmonary tuberculosis confirmed by positive sputum. While tuberculosis could be considered a type of pneumonia, we included it as a false positive given the significant difference in treatment. When evaluating patients with suspected pneumonia in a setting with high rates of tuberculosis and chronic lung parenchymal disease, it is important to consider the clinical scenario to help differentiate findings on diagnostic imaging.

Our study had a high incidence of pneumonia (71%). Other studies in developed countries evaluating ultrasound for diagnosis of pneumonia also had high rates of pneumonia, including Cortellero et al. (67.5%) and Bourcier et al. (85%) [9, 10]. The study in China by Liu et al. found pneumonia in 62.6% of their study population [8]. As a leading cause of death in Nepal, we believe this represents the Nepali patient population with high rates of pneumonia. Having an inexpensive, sensitive bedside test like lung ultrasound to assure clinician’s do not miss this diagnosis and can more confidently rule out pneumonia with a negative test could help clinicians in this resource-limited setting.

Lung ultrasound was able to be performed and interpreted rapidly at the bedside. In this setting, given that chest X-ray took an average of nearly 2 h to be performed (not including print time and physician interpretation), bedside lung ultrasound can provide a quicker diagnosis with timely and appropriate therapy. Bedside chest ultrasound is also cheaper than chest X-ray, costing on average one third to half as much as chest X-ray and, therefore, providing a more sensitive test at reduced costs [26]. Additionally, its use may provide a more patient and family-centered diagnostic approach, especially in this setting where portable imaging is not available and the family is required to take the patient to radiology, which depending on severity of illness may require an oxygen tank, intravenous fluids, and wheelchair or stretcher. Increasing awareness of the diagnostic accuracy of ultrasound for pneumonia and training clinicians could improve evaluation of patients with pneumonia throughout Nepal and other resource-limited settings.

This study has several limitations. The study was designed to evaluate a difference in sensitivity between lung ultrasound and chest X-ray, which required a sample size of 62 patients. This small sample size gives limited information regarding specificity and other diagnostic characteristics of lung ultrasound in this setting. A repeat study with a larger, more heterogeneous sample is needed to further evaluate these diagnostic characteristics, recognizing that the specificity may not perform as well in low-income countries. Also, the study was completed in one hospital in an urban city in Nepal, making it difficult to extrapolate results to other rural and resource-limited areas. Despite this, our patient population represents the general population of Nepal with its prevalence of disease. Therefore, we believe lung ultrasound would perform similarly in other settings within Nepal and settings similar to Nepal [16–18, 27]. Additionally, ultrasonography is an operator-dependent examination that requires training. The results of this paper are based on four emergency physicians trained in lung ultrasound. While the study demonstrated reliability of the ultrasound readings and subsequent results, broad implementation of lung ultrasound requires training and assessment of clinicians. There is no consensus on what level of training is required to achieve competency in lung ultrasonography for pneumonia, and the results in other settings may be different, due to different skills and experience. Despite this, these results demonstrate the benefit of including lung ultrasound in training and in implementation of guidelines for pneumonia diagnosis and treatment in Nepal and other resource-limited countries.

## Conclusions

Bedside lung ultrasound demonstrated better sensitivity than chest X-ray for the diagnosis of pneumonia in Nepal. Given this diagnostic performance, lung ultrasound could be another imaging modality used to diagnose pneumonia in a resource-limited setting.

## Abbreviations

CT: Computed tomography
PA: Posterioranterior
